# Genetic and Biological Diversity of Porcine Sapeloviruses Prevailing in Zambia

**DOI:** 10.3390/v12020180

**Published:** 2020-02-05

**Authors:** Hayato Harima, Masahiro Kajihara, Edgar Simulundu, Eugene Bwalya, Yongjin Qiu, Mao Isono, Kosuke Okuya, Gabriel Gonzalez, Junya Yamagishi, Bernard M. Hang’ombe, Hirofumi Sawa, Aaron S. Mweene, Ayato Takada

**Affiliations:** 1 Hokudai Center for Zoonosis Control in Zambia, School of Veterinary Medicine, the University of Zambia, P.O. Box 32379, Lusaka 10101, Zambia; harima@czc.hokudai.ac.jp (H.H.); yongjin_qiu@czc.hokudai.ac.jp (Y.Q.); 2 Department of Disease Control, School of Veterinary Medicine, the University of Zambia, P.O. Box 32379, Lusaka 10101, Zambia; esikabala@yahoo.com (E.S.); h-sawa@czc.hokudai.ac.jp (H.S.); asmweene04@yahoo.com (A.S.M.); 3 Department of Clinical Studies, School of Veterinary Medicine, the University of Zambia, P.O. Box 32379, Lusaka 10101, Zambia; eugenelb2000@yahoo.com; 4 Division of Global Epidemiology, Hokkaido University Research Center for Zoonosis Control, N20 W10, Kita-ku, Sapporo 001-0020, Japan; misono@czc.hokudai.ac.jp (M.I.); kokuya@czc.hokudai.ac.jp (K.O.); 5 Division of Bioinformatics, Hokkaido University Research Center for Zoonosis Control, N20 W10, Kita-ku, Sapporo 001-0020, Japan; gagonzalez@czc.hokudai.ac.jp; 6 Division of Collaboration and Education, Hokkaido University Research Center for Zoonosis Control, N20 W10, Kita-ku, Sapporo 001-0020, Japan; junya@czc.hokudai.ac.jp; 7 Global Station for Zoonosis Control, Global Institution for Collaborative Research and Education (GI-CoRE), Hokkaido University Kita-ku, Sapporo 001-0020, Japan; 8 Department of Para-Clinical Studies, School of Veterinary Medicine, the University of Zambia, P.O. Box 32379, Lusaka 10101, Zambia; mudenda68@yahoo.com; 9 Africa Center of Excellence for Infectious Diseases of Humans and Animals, the University of Zambia, P.O. Box 32379, Lusaka 10101, Zambia; 10 Division of Molecular Pathobiology, Hokkaido University Research Center for Zoonosis Control, N20 W10, Kita-ku, Sapporo 001-0020, Japan; 11 Global Virus Network, 725 West Lombard St, Room S413, Baltimore, MD 21201, USA

**Keywords:** porcine sapelovirus, prevalence, complete genome, characterization, recombination, Zambia

## Abstract

Porcine sapelovirus (PSV) has been detected worldwide in pig populations. Although PSV causes various symptoms such as encephalomyelitis, diarrhea, and pneumonia in pigs, the economic impact of PSV infection remains to be determined. However, information on the distribution and genetic diversity of PSV is quite limited, particularly in Africa. In this study, we investigated the prevalence of PSV infection in Zambia and characterized the isolated PSVs genetically and biologically. We screened 147 fecal samples collected in 2018 and found that the prevalences of PSV infection in suckling pigs and fattening pigs were high (36.2% and 94.0%, respectively). Phylogenetic analyses revealed that the Zambian PSVs were divided into three different lineages (Lineages 1–3) in the clade consisting of Chinese strains. The Zambian PSVs belonging to Lineages 2 and 3 replicated more efficiently than those belonging to Lineage 1 in Vero E6 and BHK cells. Bioinformatic analyses revealed that genetic recombination events had occurred and the recombination breakpoints were located in the L and 2A genes. Our results indicated that at least two biologically distinct PSVs could be circulating in the Zambian pig population and that genetic recombination played a role in the evolution of PSVs.

## 1. Introduction

The genus *Sapelovirus*, belonging to the family *Picornaviridae*, consists of two species: *Sapelovirus A* and *Sapelovirus B*, including porcine sapelovirus (PSV) and simian sapelovirus, respectively [1]. PSV was formerly designated porcine enterovirus 8 (PEV-8) [2]. PSV is a non-enveloped, positive-sense, single-stranded RNA virus, and its genome size is approximately 7.5 kb, consisting of a 5′ untranslated region (UTR), large open reading frame (ORF), 3′ UTR, and polyadenylation tail (poly (A) tail) [3]. The ORF encodes a single polyprotein composed of a leader protein (L), four structural proteins (VP4, VP2, VP3, and VP1), and seven non-structural proteins (2A, 2B, 2C, 3A, 3B, 3C, and 3D) [3]. VP1, VP2, and VP3 are the major capsid proteins, which are located on the surface of the virion, and the VP1 sequence is the most diverse among the encoded proteins of PSV [3,4].

Since the first detection of PSV in the UK in 1958 [5], it has been identified in many countries, including the USA, Brazil, Australia, Japan, China, South Korea, India, Germany, France, Italy, the Czech Republic, and the UK [4,6,7,8,9,10,11,12,13,14,15,16,17,18,19,20,21,22,23]. In Kenya and Uganda, a genomic fragment of PSV was detected from pig feces by metagenomic analysis [24], but the prevalence and genetic diversity of PSVs in Africa are totally unknown.

PSV infections in pigs under field conditions are often subclinical. While high prevalences of PSV infection in diarrheic pig herds were observed in Asia and Europe, PSVs were also detected frequently in asymptomatic pigs [14,16,17,19,20,21,24]. Indeed, PSV was shown to cause various symptoms such as encephalomyelitis, diarrhea, and pneumonia in experimentally infected pigs [6,7,25]. Accordingly, some outbreaks of nervous disease and gastroenteritis associated with respiratory symptoms caused by PSV infection were recently reported in the USA, UK, and China [4,6,18]. The affected pigs showed diarrhea, respiratory distress, ataxia, paresis, mental dullness, and decreased responses to environmental stimuli, and finally died within a few days after the clinical onset. While genetic diversity of PSVs has been reported [19,20], phenotypic differences among PSV strains due to genetic features have not been discussed. Little is known about the pathogenesis of PSV infection of pigs, therefore the etiological relationship between the presence of PSV and clinical symptoms is still controversial.

In this study, we investigated the prevalence of PSV infection in the pig population in Zambia and determined the full genome sequences of PSVs isolated from fecal samples. The PSV isolates were characterized genetically and biologically.

## 2. Materials and Methods

### 2.1. Sample Collection

We focused on the farms having more than 500 pigs and selected five farms where the owners agreed to sampling (Farms A and E in Lusaka, Farm B in Chilanga, Farm C in Kafue, and Farm D in Chibombo District) and samples were collected with local veterinary personnel during the period from January through December 2018 (Table 1). For the collection of fecal samples, some pens were chosen at random, and fresh stools on the floor were collected into tubes. Fecal samples were collected from suckling (0 to 3 weeks old) and fattening pigs (4 to 12 weeks old) with or without diarrhea.

### 2.2. Detection of PSV by RT-PCR

Fecal samples were suspended in phosphate-buffered saline (PBS) containing 5% antibiotic–antimycotic solution (Anti-Anti) (Life Technologies, Gibco, Waltham, MA, USA) and briefly centrifuged. Total RNAs were extracted from the supernatants using TRIzol-LS (Thermo Fisher Scientific, Waltham, MA, USA) according to the manufacturer’s instructions. RNA samples were examined for the detection of PSV genome using RT-PCR with specific primers designed based on a conserved sequence within the 5′ UTR as previously described [20] and PrimeScript One Step RT-PCR Kit Ver. 2 (Takara, Shiga, Japan) according to the manufacturer’s protocol (Supplementary Table S1). RT-PCR conditions were as follows: a cDNA synthesis step at 50 °C for 30 min, PCR activation step at 94 °C for 2 min, followed by 35 cycles at 94 °C for 30 s, 53 °C for 30 s, 72 °C for 40 s, and a final extension at 72 °C for 7 min. PCR products were separated using agarose gel electrophoresis and visualized with ethidium bromide staining under an ultraviolet illuminator.

### 2.3. Cells and Virus Isolation

African green monkey kidney cells (Vero E6) and baby hamster kidney (BHK) cells were used in this study. These cells were maintained in Dulbecco’s Modified Eagle’s Medium (D-MEM) (Nissui Pharmaceutical Co., Tokyo, Japan) supplemented with 10% fetal bovine serum (FBS), 2 mM l-glutamine, 100 units/mL penicillin, 100 µg/mL streptomycin, 3.5 mg/mL D-glucose, and 1.0 mg/mL NaHCO3 at 37 °C with 5% CO_2_.

The supernatants of fecal suspensions were inoculated into Vero E6 and BHK cells, followed by one-hour incubation at 37 °C with 5% CO_2_ for virus adsorption. After the inocula were removed, the cells were washed twice with PBS and maintained in Eagle’s minimum essential medium (MEM) (Nissui Pharmaceutical Co., Tokyo, Japan) containing 2% FBS, 2 mM l-glutamine, 4% Anti-Anti, and 1.0 mg/mL NaHCO_3_ at 37 °C with 5% CO_2_. The supernatants of the Vero E6 and BHK cells were passaged three times to observe cytopathic effects (CPE). The cultured media collected from the cells showing CPE were filtered through 0.45 µm pore membrane filters (IWAKI, Tokyo, Japan) to remove bacterial cells, and re-inoculated into the respective cell lines. Isolation of a single PSV strain was confirmed by RT-PCR and next generation sequencing. We further verified that there were no other viruses in the supernatants by next generation sequencing.

### 2.4. Whole Genome Sequencing

Cultured media were harvested from cells showing CPE and briefly centrifuged to remove cell debris. Total RNAs were extracted from the supernatants using QIAamp Viral RNA Mini Kits (Qiagen, Hilden, Germany) and cDNAs were synthesized using a PrimeScript Double Strand cDNA Synthesis Kit (Takara, Shiga, Japan) according to the manufacturer’s instructions. The double-stranded cDNA were fragmented and index adapters were attached using a Nextera XT DNA Library Preparation Kit (illumina, San Diego, CA, USA) according to the manufacturer’s instructions. The libraries were subjected to whole genome sequencing on a MiSeq instrument with a MiSeq Reagent Kit v3 (600 cycles) (Illumina, San Diego, CA, USA). Sequence data were analyzed using CLC Genomics Workbench software (CLC bio, Qiagen, Hilden, Germany). After trimming of low-quality reads, remaining reads were *de novo* assembled using the default setting. Obtained contigs were analyzed by local BLAST with virus references downloaded from the NCBI database to identify virus species. Consensus sequences obtained by mapping to the PSV reference sequences (GenBank accession nos. KJ463384 and KX574284) were aligned with the PSV contigs of *de novo* assembly. Finally, all trimmed reads were re-mapped to the aligned PSV sequences and consensus sequences with coverage of over 20 reads were obtained. Additionally, the 5′ and the 3′ terminal regions of the complete genome and the VP1 C-terminal regions were amplified by RT-PCR and the rapid amplification of cDNA ends (RACE) method with PSV-specific primers to determine the sequences of low-coverage regions by Sanger sequencing (Supplementary Table S1). RT-PCR and RACE were performed with a PrimeScript One Step RT-PCR Kit Ver. 2 (Takara, Shiga, Japan) and a SMARTer RACE cDNA Amplification Kit (Takara, Shiga, Japan) according to the manufacturer’s protocol. PCR products purified from agarose gels were subjected to direct sequencing using a BigDye Terminator v3.1 Cycle Sequencing kit (Life Technologies, Applied Biosystems, Foster City, CA, USA) with ABI PRISM 3130 (Life Technologies, Applied Biosystems, Foster City, CA, USA).

### 2.5. Phylogenetic Analysis and Other Bioinformatic Analyses

The complete ORF and cleavage sites of PSV were predicted by comparing with the genome of previously known PSV strains. Bioinformatic analyses were performed with various PSV sequences obtained from the DDBJ/EMBL-Bank/GenBank database (Supplementary Table S2). Phylogenetic analyses of the complete ORF, VP1, and 3C + 3D (3CD) nucleotide sequences were performed using PhyML 3.0 web server with akaike information criterion [26,27]. The MUSCLE software was used to align the sequences, and phylogenetic trees were constructed using the maximum likelihood method with the GTR + G + I model and 1000 bootstrap replications. The amino acid sequences of the VP1 C-terminal region and polyprotein were aligned using MUSCLE. Global identity analyses were conducted between Zambian and other representative PSVs using GENETYX version 12 (GENETYX Corporation, Tokyo, Japan). Recombination analyses were conducted using Simplot software version 3.5.1 [28] and Recombination Detection Program (RDP) version 4.97 [29].

### 2.6. Growth Kinetics of PSV

PSVs isolated using Vero E6 and BHK cells were propagated in Vero E6 and BHK cells, respectively. Plaque forming units (pfu) of stock viruses were determined using Vero E6 cells. Fifty-percent tissue culture infectious doses (TCID_50_) were determined using BHK cells since obvious plaque formation was not observed in BHK cells infected with PSVs. For the plaque assay, confluent monolayers of Vero E6 cells were infected with 10-fold serially diluted viruses. After adsorption for one hour, the inocula were removed and the cell monolayers were overlaid with MEM containing 2% FBS, 2 mM l-glutamine, 100 units/mL penicillin, 100 µg/mL streptomycin, 1.0 mg/mL NaHCO_3_, and 1% agar. Plaque-forming units were determined two days post-infection. For the TCID_50_ assay using four wells per dilution, confluent monolayers of BHK cells were infected with 10-fold serially diluted viruses. CPE was observed three days post-infection and the viral titers were calculated by the Reed-Muench method [30].

Vero E6 and BHK cells were infected with each of the PSV isolates at a multiplicity of infection (MOI) of 0.01. After adsorption for one hour, the inocula were removed, and the cells were incubated with MEM containing 2% FBS, 2 mM l-glutamine, 100 units/mL penicillin, 100 µg/mL streptomycin, and 1.0 mg/mL NaHCO_3_ at 37 °C with 5% CO_2_. The cultured media of infected Vero E6 cells were harvested at 8, 24, 48, and 72 h post-infection, and those of infected BHK cells were harvested at 8, 24, 48, 72, and 96 h post-infection. These samples were centrifuged at 3000 rpm at 4 °C for 5 min to remove the cell debris. Viral titers of the supernatants were determined by the plaque and the TCID_50_ assays with Vero E6 and BHK cells, respectively.

### 2.7. Statistical Analysis

Differences in the prevalence rates of PSV infection were analyzed using the chi-square test. *P* values of <0.05 were considered statistically significant.

## 3. Results

### 3.1. Prevalence of PSV Infection in Zambia

In 2018, 47 and 100 fecal samples were collected from suckling and fattening pigs in Zambia, respectively. Of these samples, 40 and 59 samples were obtained from diarrheal suckling and fattening pigs, respectively (Table 2). RNA was extracted from these samples and tested by RT-PCR with specific primers for the 5′ UTR of the PSV genome (Supplementary Table S1). The PSV genome was detected in 17 of the 47 samples from suckling pigs (36.2%). Of these RT-PCR-positive samples, 42.9% (3/7) and 35.0% (14/40) were obtained from healthy and diarrheic suckling pigs, respectively (Table 2 and Supplementary Table S3). Statistical significance was not found for the positive ratios between healthy and diarrheic suckling pigs (*P* = 0.69). Remarkably high PSV prevalence was found in fattening pigs; 94 of the 100 samples (94.0%) were positive for the PSV genome. The viral genome was detected in 95.1% (39/41) and 93.2% (55/59) of healthy and diarrheal samples, respectively (Table 2 and Supplementary Table S3). There was no significant difference in the prevalence between healthy and diarrheic fattening pigs (*P* = 0.69). It was noteworthy that fattening pigs had significantly higher PSV prevalence (94.0%) than suckling pigs (36.2%) (*P* < 0.01).

### 3.2. Isolation and Full Genome Sequence of PSVs

**Figure 1:**
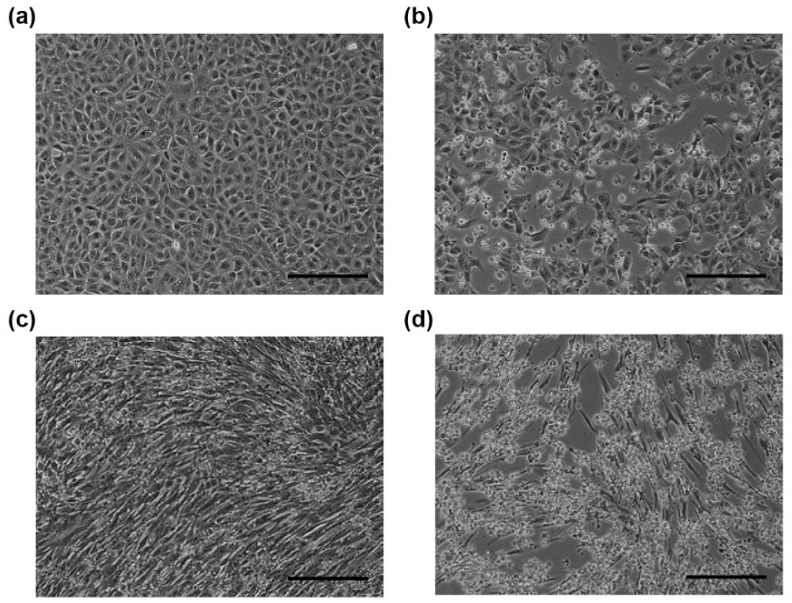
Microscopic images of African green monkey kidney cells (Vero E6) (**a**) and (**b**) and baby hamster kidney (BHK) (**c**) and (**d**) cells. Mock-infected cells (**a**) and (**c**) and PSV-infected cells (**b**) and (**d**) were observed at 24 h post-infection. The cells infected with PSV-20-V (**b**) or PSV-26-B (**d**) showed cytopathic effects. Scale bars represent 200 µm.

For virus isolation, suspensions of 111 PSV RT-PCR-positive fecal samples were inoculated into both Vero E6 and BHK cells and obvious CPE was observed in the inoculated cells (Figure 1, Supplementary Figure S1). Finally, 8 and 25 isolates were obtained from the inoculated Vero E6 and BHK-21 cells, respectively, suggesting that BHK cells were more sensitive to PSV infection. Six isolates (PSV-20, PSV-21, PSV-22, PSV-23, PSV-26, and PSV-46) were selected as representatives according to the phylogenic positions (see below) and used for further analyses (Table 3). PSV-20 was isolated only from Vero E6 cells (strain PSV-20-V), whereas PSV-22 and PSV-26 were isolated only from BHK cells (strains PSV-22-B and PSV-26-B, respectively). PSV-21 (strains PSV-21-V and PSV-21-B), PSV-23 (strains PSV-23-V and PSV-23-B), and PSV-46 (strains PSV-46-V and PSV-46-B) were isolated from both cell lines.

To obtain full genome sequences of the viruses, DNA libraries prepared from total RNA extracted from each isolate were subjected to next-generation sequencing. Through bioinformatic analyses, PSV genome sequences of more than 7000 nucleotides in length with sufficient coverage were obtained. Sanger sequencing for amplicons of RT-PCR and RACE-PCR was also performed to confirm the sequences of low-coverage regions (i.e., the 5′ and 3′ termini and the VP1 C-terminal region). Finally, we obtained complete viral genome sequences consisting of 7458–7498 nucleotides with a poly (A) tail (Table 3). The complete genome sequences of the Zambian PSVs were deposited in the DNA Data Bank of Japan (DDBJ) under accession numbers, DDBJ/EMBL/GenBank LC508226-LC508234.

### 3.3. Comparative Genome Analysis among PSVs

The complete ORF sequences of the PSV isolates were predicted by comparative analyses with other known PSVs. The amino acid sequences were then deduced by alignment with the amino acid sequence of the V13 strain (GenBank accession no. AF406813) (Supplemental Table S4). The ORF size of 4 isolates, PSV-21, PSV-22, PSV-23, and PSV-46, was 6969 nucleotides. PSV-20 and PSV-26 had longer ORFs, 6996 and 6993 nucleotides, respectively (Table 3). An alignment of Zambian and other known PSVs revealed that PSV-20 and PSV-26 had 8 and 9 amino acid insertions at C-terminus of VP1, respectively (Supplementary Figure S2). These insertions are also present in other strains isolated in China, South Korea, Japan, USA, France, and Germany, and are common among some PSV strains. Identity comparisons of ORFs among PSVs demonstrated that Zambian PSVs had 83.3%–90.6% and 92.6%–98.5% identity in nucleotide and amino acid sequences, respectively, with PSV strains detected in other continents (Supplemental Table S5). All 6 Zambian PSVs were most closely related to Chinese strains. Among Zambian PSVs, identity of nucleotide and amino acid sequences ranged from 85.6%–99.5% and 93.7%–99.7%, respectively. While PSV-20, PSV-21, PSV-22, and PSV-23 were isolated at the same farm in Lusaka in January, PSV-20 had relatively low identity of nucleotide (89.5%–89.6%) and amino acid sequences (95.1%–95.3%) with the other three isolates. PSV-20 showed rather higher identity with PSV-26 found on another farm in Chilanga. These data indicated that more than two genetically distinct PSVs were simultaneously circulating on a single farm. Sequence identity of the viral proteins were also compared among Zambian PSVs (Supplemental Table S6). VP1, a capsid protein, sequence had the highest diversity among 12 viral proteins with the amino acid sequence identity of the protein ranging from 78.9–99.6%. In contrast, nonstructural proteins such as 2B, 2C, 3A, 3C, and 3D were well conserved, and the range of amino acid identities was 97.0%–100%.

### 3.4. Phylogenetic Analyses of PSVs

**Figure 2:**
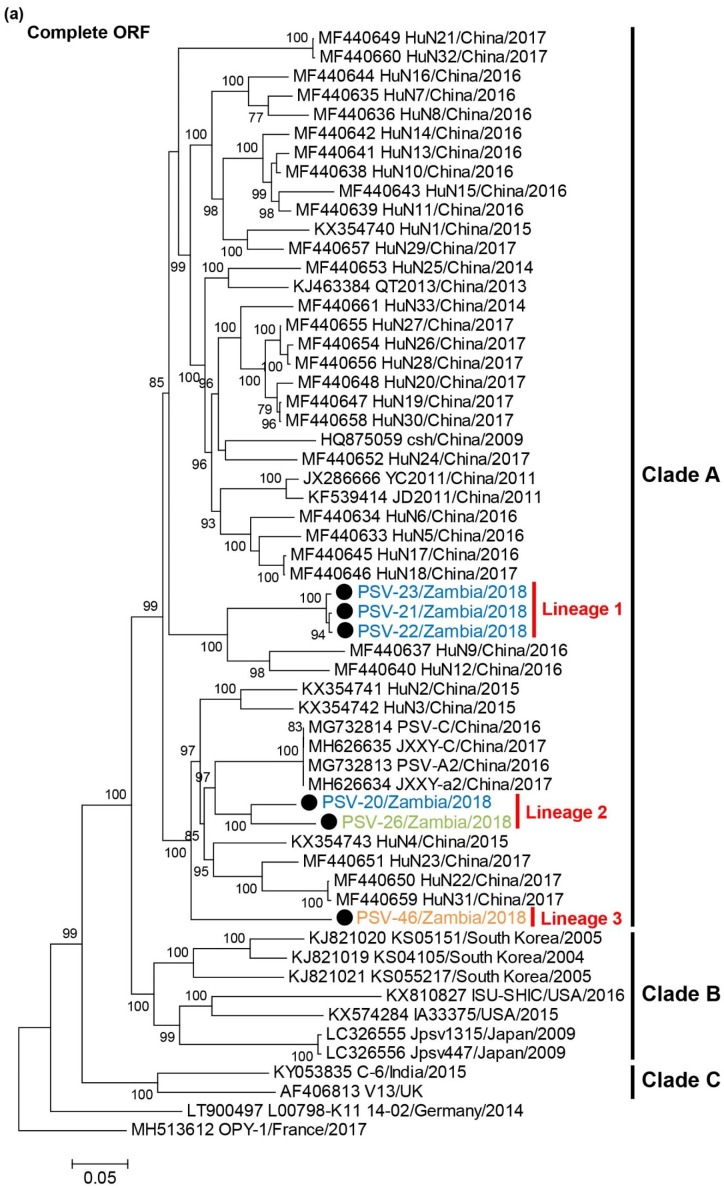

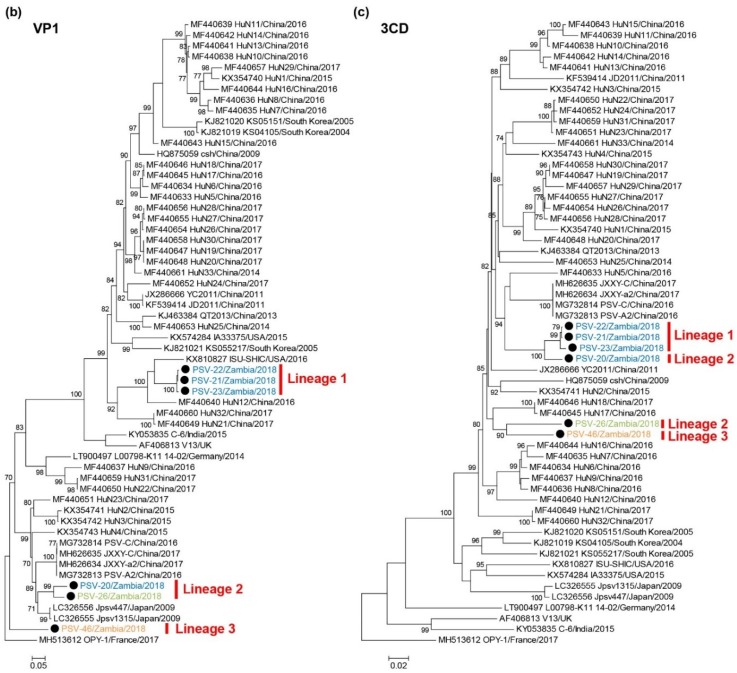
Phylogenetic analysis of PSVs. Based on 6969–7014 nucleotide complete open reading frame (ORF) sequences (**a**), 855–894 nucleotides for VP1 (**b**), and 1929-1932 nucleotides for 3CD (**c**), the trees were constructed using the maximum likelihood method with the GTR + G + I model and 1000 bootstrap replications. Bootstrap values greater than 70% are shown on the interior branch nodes, and clades and lineages are indicated on the tree. The scale bar indicates the number of substitutions per site. Black circles represent the PSVs isolated in this study. Zambian strain names are labeled with different colors as follows: PSV-20, PSV-21, PSV-22, and PSV-23 isolated in Farm A are in blue, PSV-26 isolated in Farm B is in green, PSV-46 isolated in Farm D is in orange.

A phylogenetic tree based on complete ORF nucleotide sequences was constructed using the sequence data of Zambian and other representative PSVs retrieved from GenBank (Figure 2a, Supplementary Table S2). PSVs were phylogenetically divided into three clades (Clade A to C). Clade A exclusively included Chinese strains. Clade B consisted of strains detected in Japan, the USA and South Korea. Clade C were composed of the UK and Indian strains. Two European strains did not belong to these clades. All Zambian PSVs were grouped into Clade A. Zambian PSVs were further divided into three different lineages, Lineage 1 (PSV-21, PSV-22, and PSV-23), Lineage 2 (PSV-20 and PSV-26), and Lineage 3 (PSV-46).

Phylogenetic analyses of the VP1 and 3CD nucleotide sequences were also conducted. In the phylogenetic tree based on the VP1 gene, the Zambian PSVs were divided into three lineages, Lineages 1, 2, and 3, in the same manner as the tree of complete ORFs (Figure 2b). PSV-20 and PSV-26 clustered with Clade B strains such as Jpsv447 and Jpsv1315, and PSV-46 formed an individual branch. The 3CD-based tree showed that Zambian PSVs were phylogenetically separated into two different groups and formed a monophyletic clade with Clade A strains. PSV-20 was more closely related to the Lineage 1 viruses than the Lineage 2 (i.e., PSV-26) virus (Figure 2c). These results suggested that PSV-20, PSV-26, and PSV-46 had undergone genetic recombination between genetically distinct PSVs.

### 3.5. Recombination Analyses of PSVs

**Figure 3:**
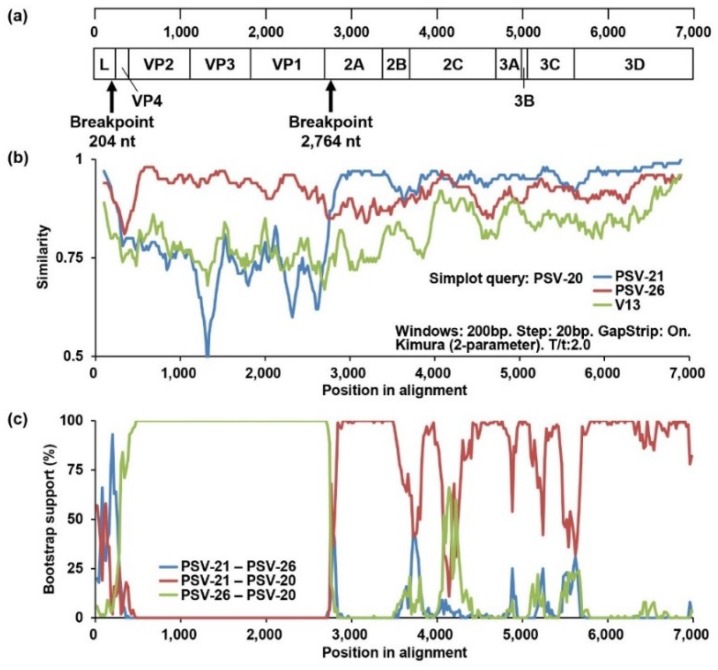
Identification of the recombination events among Zambian PSVs. (**a**) Predicted recombination breakpoints are shown with black arrows on the schematic diagram of the PSV genome. (**b**) Similarity analyses of complete ORF nucleotide sequences of PSV-20, PSV-21, PSV26, and V13 strains were performed using SimPlot software version 3.5.1 with the default setting. The genome of PSV-20 was used as a query sequence. The similarities plots of PSV-21 (blue line), PSV-26 (red line), and V13 (green line) are shown. (**c**) Recombination breakpoint analyses were conducted using RDP version 4.97 with the sequences of all Zambian PSVs. The recombination among PSV-20, PSV-21, and PSV-26 predicted by the analyses using the Bootscan method is shown. The bootstrap support values of PSV-21 versus PSV-26 (blue line), PSV-21 versus PSV-20 (red line), and PSV-26 versus PSV-20 (green line) are shown.

To obtain evidence of genetic recombination in Zambian PSVs, recombination analyses were conducted using SimPlot software and RDP. Since the phylogenetic position of PSV-20 was inconsistent among the full-length ORF, VP1, and 3CD trees, we performed a similarity analysis using Simplot software with PSV-20, PSV-21 (Lineage 1), and PSV-26 (Lineage 2) sequences. A UK strain, V13, was used as an outside group strain. PSV-20 had high nucleotide sequence similarity with PSV-26 in the VP4, VP2, VP3, and VP1 regions, whereas the 2A, 2B, 2C, 3A, 3B, 3C, and 3D sequences were highly similar to PSV-21 sequences (Figure 3b). Next, recombination breakpoint analyses for all Zambian PSVs were performed using RDP version 4.97 with seven different methods, RDP, GeneConv, BootScan, MaxChi, Chimaera, SiScan, and 3Seq. The analyses also predicted genetic recombination with combinations of PSV-20/PSV-21 and PSV-20/PSV-26 in all algorithms. Bootscanning analysis showed potential recombination breakpoints located in the L and 2A regions (*P* = 1.031 × 10^−31^) (Figure 3a,c), indicating that PSV-20 had Lineage 2-like capsid proteins, VP1, VP2, VP3, and VP4, in the genetic backbone of Lineage 1 viruses. These findings suggested that PSV-20 might evolve from an ancestor that emerged through genetic recombination between Lineage 1 and 2 viruses. However, in the recombination analyses with the whole alignment used for the phylogenetic tree shown in Figure 2a, the Jpsv447 strain isolated in Japan was suggested to be the genetically closest strain to a potential ancestor instead of PSV-26 although a similar genetic recombination profile with the potential recombination breakpoints locating in the L and 2A regions between PSV-20 and Jpsv447 was found (*P* = 1.672 × 10^−39^) (Supplemental Figure S3). It was also predicted that PSV-26 was also a recombinant virus and that this region of PSV-20, PSV-26, and Jpsv447 descended from the common ancestor (Supplemental Figures S3 and S4). Although breakpoints and potential parents could not be determined most likely due to low *P* values, PSV-46 was also predicted as a recombinant virus (Supplemental Table S7).

### 3.6. Growth Kinetics of Zambian PSVs In Vitro

**Figure 4:**
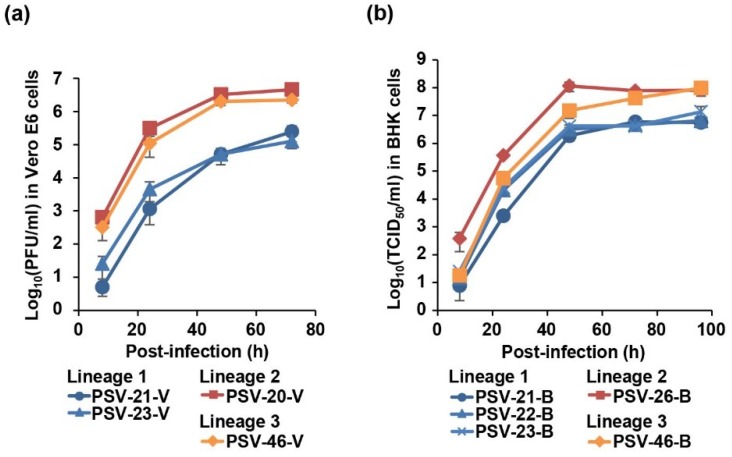
Growth kinetics of Zambian PSVs in cell culture. (**a**) Vero E6 cells were infected with PSV-20-V, PSV-21-V, PSV-23-V, and PSV-46-V at multiplicity of infection (MOI) 0.01. (**b**) BHK cells were infected with PSV-21-B, PSV-22-B, PSV-23-B, PSV-26-B, and PSV-46-B at MOI 0.01. The supernatants were harvested at the indicated time points and viral titers were determined by plaque assay for Vero-E6 cells and fifty-percent tissue culture infectious dose (TCID_50_) assay for BHK cells. Each value represents the mean ± standard error of the results of three independent experiments.

To biologically characterize Zambian PSVs, we analyzed the viral growth kinetics in cell culture. To remove influences of adaptive mutations acquired through the virus isolation, the growth kinetics were assessed in the same cell lines used for virus isolation. Vero E6 cells were infected with PSV-20-V, PSV-21-V, PSV-23-V, and PSV-46-V (Figure 4a) and BHK cells were infected with PSV-21-B, PSV-22-B, PSV-23-B, PSV-26-B, and PSV-46-B (Figure 4b) at MOI 0.01. Virus titers of all Zambian PSVs reached an almost plateau level at 48 h post-infection in both cell lines (Figure 4). Zambian PSVs belonging to Lineages 2 and 3 showed approximately 10- to 100-fold higher titers than Lineage 1 viruses in Vero E6 cells (Figure 4a). Similarly, Lineage 2 and 3 viruses also had approximately 10-fold higher titers than Lineage 1 viruses in BHK cells (Figure 4b). These results suggested that Zambian PSVs had different biological phenotypes depending on their genetic lineages.

## 4. Discussion

Although PSV is one of the viruses commonly distributed in pigs throughout Europe, Asia, Australia, and North America to South America [6,7,8,9,11,13,14,15,16,17,18,19,20,21,22], the prevalence and geographical distribution of PSV infection and genetic diversity of PSVs in Africa are totally unclear. The only available report on Africa is on the detection of a partial PSV genome fraction in asymptomatic pigs via a metagenomic approach [24]. In this study, we demonstrated that PSV infection was prevalent in the pig population in Zambia. In our screening, PSV genome prevalence was higher in fattening pigs (94.0%) than in suckling pigs (36.2%) (Table 2). This pattern is also observed in the pig populations in the Czech Republic and China [17,20]. It may be that the higher susceptibility of grown pigs is due to maternal antibody waning [31]. Alternatively, pigs may become PSV carriers via persistent infection. However, it is quite difficult to reasonably explain this higher PSV prevalence in fattening pigs since the long-term clinical course of PSV infection, including the duration of virus shedding, is still totally unknown.

PSVs are phylogenetically classified into distinct clusters correlated with their geographical locations [7,19]. The phylogenetic analysis based on the full-genome sequences of PSVs demonstrated that Zambian PSVs formed three different lineages, Lineages 1, 2, and 3, in Clade A (Figure 2a). Since this clade had only Chinese strains until this report [7], it is conceivable that Zambian PSVs originated from Chinese strains. Though farmers in Zambia generally trade their pigs domestically and rarely introduce new pigs through international trade, it is possible that PSVs prevalent in China were accompanied international human movement, resulting in the spread of Chinese PSVs into Zambia. Actually, there are reports that OIE-listed pathogens such as highly pathogenic avian influenza virus and African swine fever virus, were detected in meat products brought by international passengers from China [32,33,34]. However, the genetic data of PSVs are too limited to give a reasonable interpretation for the global transmission pattern and the evolutionary history of PSVs. Increased epidemiological and sequence data are required to better understand the evolution of PSVs.

Generally, recombination, deletion, insertion, and point mutation are the major modes of genetic and phenotypic evolution of non-segmented viruses [35]. Recently, evidence of recombination among PSVs has been reported in Japan and China, and the possible recombination breakpoints were predicted to be in an upstream region of the 2B gene [19,20]. In the present study, some genetic recombinant viruses including PSV-20, PSV-26, and PSV-46 were isolated in Zambia. The phylogenetic analyses suggest that the capsid proteins of PSV-20 and PSV-26 are derived from the same Clade B ancestor, while their non-structural genes have been acquired from two different Clade A strains. On the other hand, the capsid proteins of PSV-26 and PSV-46 might be derived from different parental strains, while their non-structural genes likely originated from the same ancestral Clade A strain (Figure 2). Recombination analyses predicted that the most probable recombination breakpoints of PSV-20 and PSV-26 were located in the L and 2A regions although breakpoints of PSV-46 could not be determined (Figure 3). These results indicate the genetic diversity of Zambian PSVs evolving by several recombination events. Although PSV has been considered to be non- or low-pathogenic for pigs, some outbreaks of various severe illness caused by PSV infection were recently reported in multiple countries [4,6,18]. Thus, it might be possible that PSVs acquire higher pathogenicity through genetic recombination.

We found that the Lineage 2 and 3 viruses more efficiently grew in vitro than the Lineage 1 viruses (Figure 4). Most of the amino acid residues unique to the Lineage 1 viruses were found in the capsid proteins; i.e., VP1, VP2, VP3, and VP4 (81 of 84 amino acid residues) (Supplementary Table S8). VP1, VP2, and VP3 are the major capsid proteins located on the virion surface and VP4 faces the inside of the capsid [3]. These capsid proteins cooperatively mediate essential steps of the virus life cycle such as entry, release of the virus genome into infected cells, and viral assembly and release [36,37,38]. Therefore, it is reasonable to hypothesize that these capsid proteins are responsible for efficient viral replication in vitro and potentially viral pathogenicity. The finding that PSV-20, which has the Lineage 2-capsid proteins in the Lineage 1-backbone, showed higher growth ability in vitro also supports this hypothesis.

It is still controversial whether natural PSV infection of farm pigs causes illness such as diarrhea. In the present study, PSVs were detected in both diarrheal and non-diarrheal pigs (Table 2). Actually, it has been frequently reported that PSVs are detected in asymptomatic pigs [14,16,17,19,20,21,24]. However, experimental PSV infections have undoubtedly induced diarrhea, encephalomyelitis, and pneumonia in pigs [6,7,25]. Indeed, lethal PSV infections of 3- to 12-week-old pigs with nervous disorders, gastroenteritis, and respiratory diseases were reported in the USA, UK, and China [4,6,18]. Porcine circovirus-2 is also universally distributed in the pig population worldwide. While porcine circovirus-2 infection itself is asymptomatic, infected pigs occasionally develop severe postweaning multisystemic wasting syndrome upon viral co-infection or immunological stimulation [39]. Similarly, PSV may also require unidentified factors to show full pathogenicity in pigs. To properly estimate the negative impact of PSV infection on the pig industry, further studies are required to better understand the epidemiology of PSV infection and the molecular mechanisms underlying its pathogenicity.

## Figures and Tables

**Table 1:** Summary of sampling in 2018 in Zambia.

Farm	District	Date	No. of Fecal Samples
A	Lusaka	7 January, 14 June	39
B	Chilanga	25 January, 10 July, 4 December	30
C	Kafue	13 February, 8 June, 16 August	58
D	Chibombo	2 March	4
E	Lusaka	17 July, 20 December	16

**Table 2:** Prevalence of porcine sapelovirus (PSV) infection of pigs in Zambia.

Age (Week)	Stage	No. of Positive/No. of Tested Samples (%)		
		Healthy	Diarrheal	Total
0–3	Suckling	3/7 (42.9)	14/40 (35.0)	17/47 (36.2)
4–12	Fattening	39/41 (95.1)	55/59 (93.2)	94/100 (94.0)

**Table 3:** Summary of Zambian PSVs whose full genome sequences were determined.

Strain	Farm	District	Sampling Date	Cell Line ^1^	Obtained Genome Length (nt) ^2^	Length of Complete ORF (nt)
PSV-20-V	A	Lusaka	7 January	Vero E6	7495	6999
PSV-21-V	A	Lusaka	7 January	Vero E6	7458	6972
PSV-21-B	A	Lusaka	7 January	BHK	7468	6972
PSV-22-B	A	Lusaka	7 January	BHK	7468	6972
PSV-23-V	A	Lusaka	7 January	Vero E6	7462	6972
PSV-23-B	A	Lusaka	7 January	BHK	7462	6972
PSV-26-B	B	Chilanga	25 January	BHK	7493	6996
PSV-46-V	D	Chibombo	2 March	Vero E6	7469	6972
PSV-46-B	D	Chibombo	2 March	BHK	7498	6972

^1^ Used for virus isolation. ^2^ Edited out poly (A) tail. Abbreviation: nucleotide, nt.

## Supplementary Materials

The following are available online at https://www.mdpi.com/1999-4915/12/2/180/s1, Table S1: Primers used in this study, Table S2: Reference sequences used in this study, Table S3: Summary of the history and prevalence of PSV infection in pigs in Zambia, Table S4: Deduced amino acid sequence lengths of encoded proteins in Zambian PSVs, Table S5: Identity comparison of complete ORFs between PSVs, Table S6: Identity comparison of encoded proteins among Zambian PSVs, Table S7: Predicted recombination in Zambian PSVs, PSV-20, PSV-26, and PSV-46, Table S8: Amino acid residues unique to the Lineage 1, Figure S1: Isolation of Zambian PSV with Vero E6 and BHK cells, Figure S2: Amino acid alignment of the VP1 C-terminal region, Figure S3: Identification of the recombination events among PSV-20, PSV-21, and Jpsv447, Figure S4: Identification of the recombination events among PSV-22, PSV-26, and Jpsv447.
