# Supplementary material for: Genetic and Biological Diversity of Porcine Sapeloviruses Prevailing in Zambia
Source: Viruses. 2020 Feb 5;12(2):180. doi: 10.3390/v12020180 (PMC7077239; doi:10.3390/v12020180)
Supplement: Supplementary file 1 [file viruses-12-00180-s001.zip › supplemental figures.pdf]

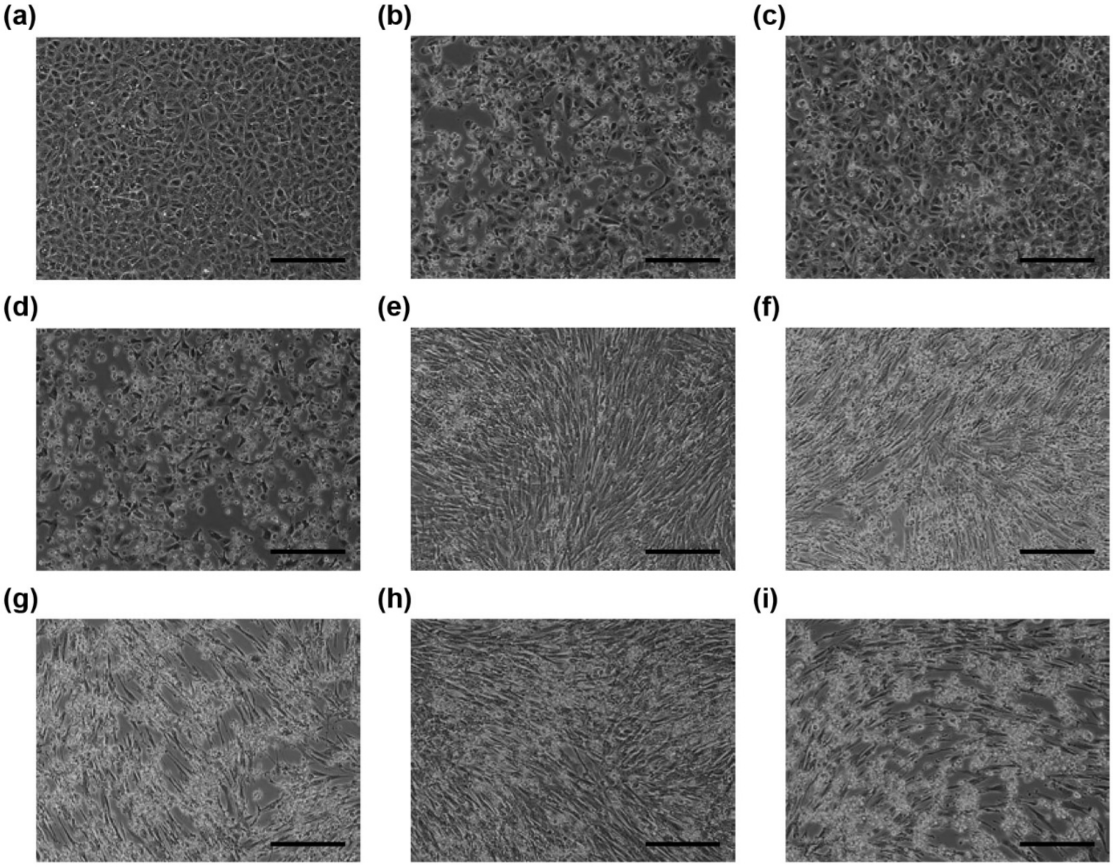

Figure S1. Microscopic images of Vero E6 (a-d) and BHK (e-i) cells. Mock-infected cells (a and e) and PSV-infected cells (b-d and f-i) were observed at 24 h post-infection. The cells infected with PSV-21-V (b), PSV-23-V (c), PSV-46-V (d), PSV-21-B (f), PSV-22-B (g), PSV-23-B (h) and PSV-46-B (i) showed cytopathic effects. Scale bars represent 200 μm.

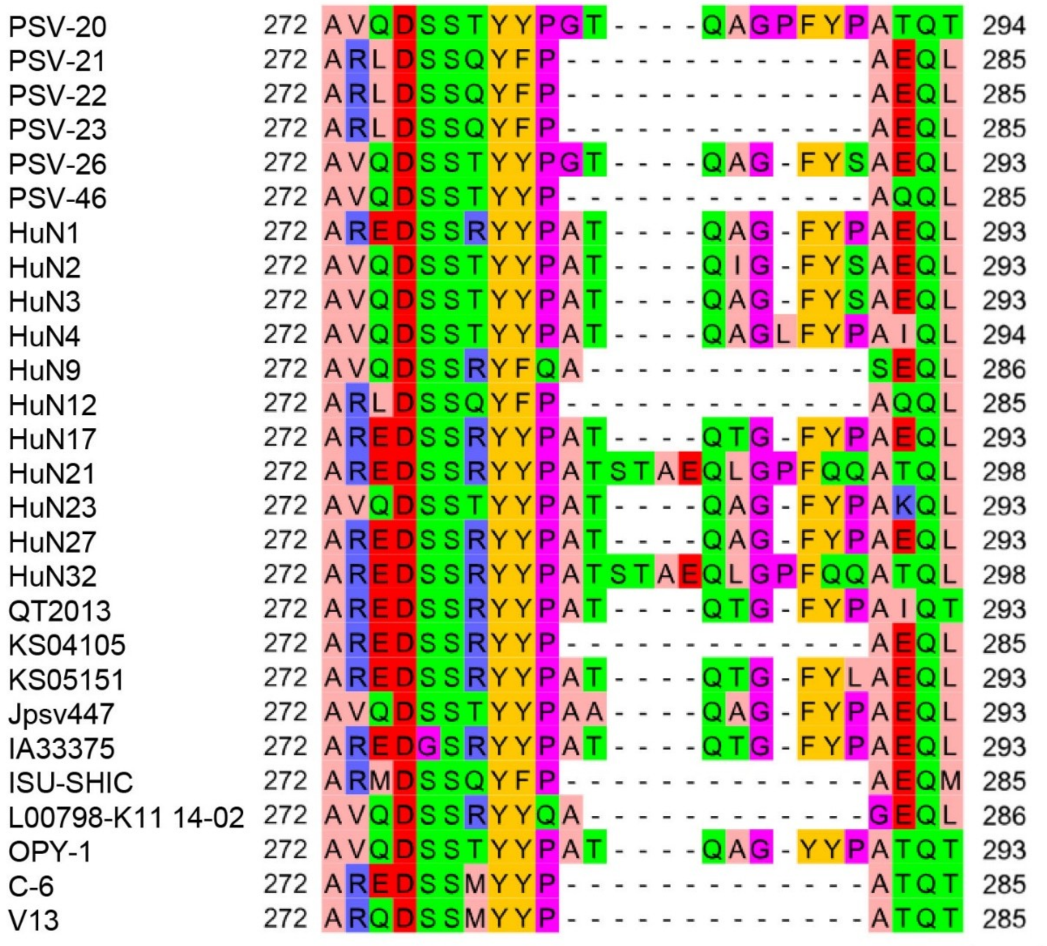

Figure S2. Amino acid alignment of the VP1 C-terminal region. The deduced amino acid sequence of the VP1 C-terminal regions of Zambian PSVs and representative PSVs were aligned. The alignment of position 272-end of the C-terminal VP1 sequences is indicated.

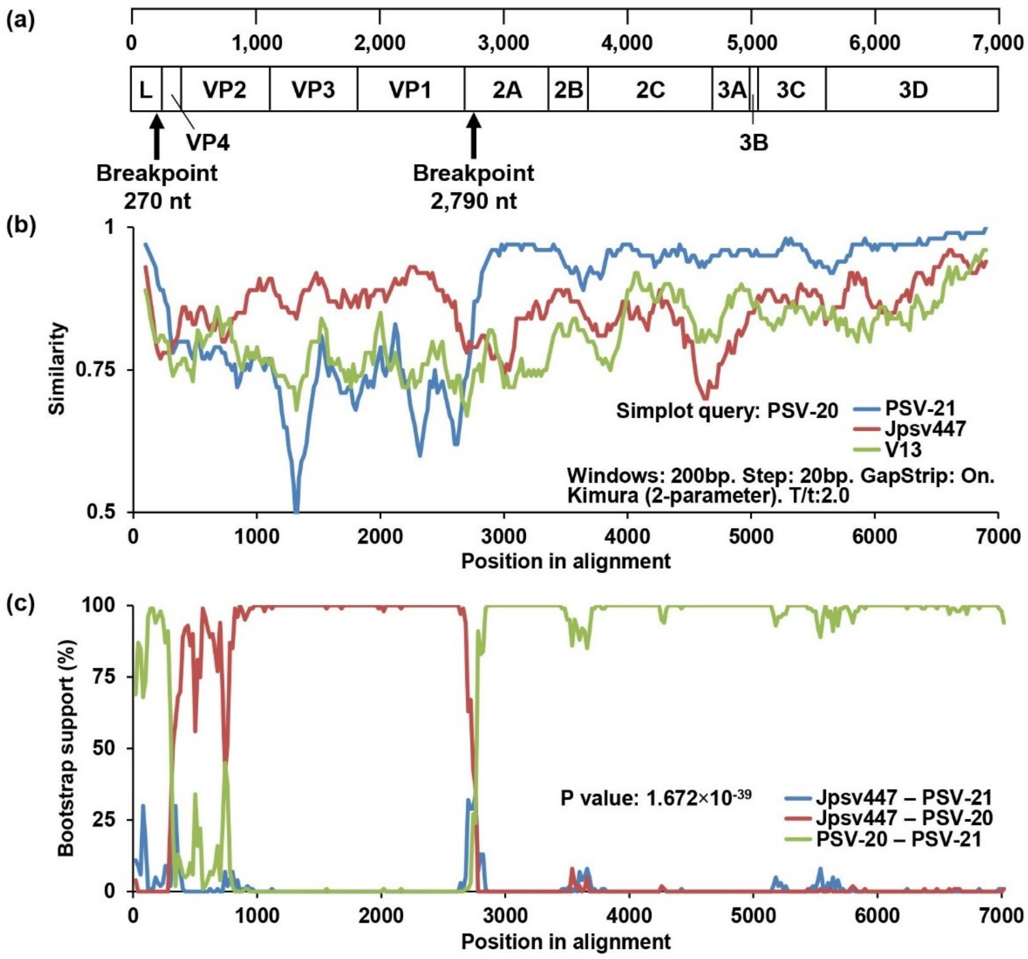

Figure S3. Identification of the recombination events among PSV-20, PSV-21, and Jpsv447. (a) Predicted recombination breakpoints are shown with black arrows on the schematic diagram of the PSV genome. (b) Similarity analyses of complete ORF nucleotide sequences of PSV-20, PSV-21, Jpsv447, and V13 strains were performed using SimPlot software version 3.5.1 with the default setting. The genome of PSV-20 was used as a query sequence. The similarities plots of PSV-21 (blue line), Jpsv447 (red line), and V13 (green line) are shown. (c) Recombination breakpoint analyses were conducted using RDP version 4.97 with the sequences of Zambian strains and reference strains. The recombination among PSV-20, PSV-21, and Jpsv447 predicted by the analyses using the Bootscan method is shown. The bootstrap support values of Jpsv447 versus PSV-21 (blue line), Jpsv447 versus PSV-20 (red line), and PSV-20 versus PSV-21 (green line) are shown.

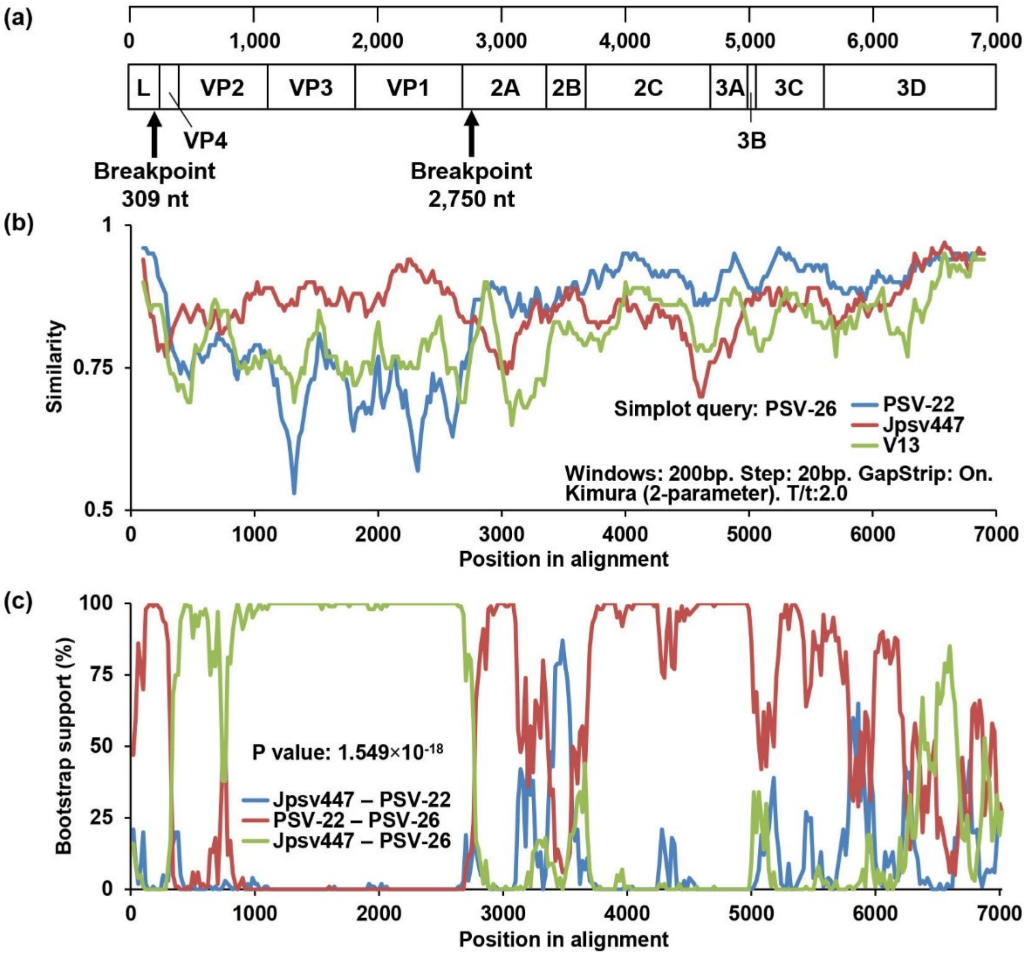

Figure S4. Identification of the recombination events among PSV-22, PSV-26, and Jpsv447. (a) Predicted recombination breakpoints are shown with black arrows on the schematic diagram of the PSV genome. (b) Similarity analyses of complete ORF nucleotide sequences of PSV-26, PSV-22, Jpsv447, and V13 strains were performed using SimPlot software version 3.5.1 with the default setting. The genome of PSV-26 was used as a query sequence. The similarities plots of PSV-22 (blue line), Jpsv447 (red line), and V13 (green line) are shown. (c) Recombination breakpoint analyses were conducted using RDP version 4.97 with the sequences of Zambian strains and reference strains. The recombination among PSV-22, PSV-26, and Jpsv447 predicted by the analyses using the Bootscan method is shown. The bootstrap support values of Jpsv447 versus PSV-22 (blue line), PSV-22 versus PSV-26 (red line), and Jpsv447 versus PSV-26 (green line) are shown.
