# Supplementary material for: Genetic and Biological Diversity of Porcine Sapeloviruses Prevailing in Zambia
Source: Viruses. 2020 Feb 5;12(2):180. doi: 10.3390/v12020180 (PMC7077239; doi:10.3390/v12020180)
Supplement: Supplementary file 1 [file viruses-12-00180-s001.zip › supplemental tables.pdf]

Table S1 Primer used in this study

Primer name	Sequence (5'-3')	Position[1]	Genomic region	Expected size (bp)	Reference
PSV-Fp	CGTGCTCCTTTGGTGATTC	156-174	5'UTR	269	[20]
PSV-Rp	GAAAGAGTAGTAGTAGATTCC	404-424			
PSV-VP1-F	TCCTTTTATGATGGTGATGG	2,829-2,847	VP1-2A	467	In this study
PSV-VP1-R	CTCCAGAGGAAAACATGTGT	3,276-3,295			
PSV-3TR-F	GATTTATTGGTTGCCTATCC	6,993-7,012	3D-3'UTR	439	In this study
PSV-3TR-R	TGTTAATCCTGTATCACTG	7,413-7,431			
PSV-5RACE-R	gattacgccaagcttGCCGCGACCCTATCAGGTAGTATAGG	337-362	5'UTR	-	In this study
PSV-5RACE-R2	gattacgccaagcttTCCCAACCTGGAAGCTCACTATGG	300-323			
PSV-3RACE-F	gattacgccaagcttTGGACCAAGAATGCCAGCTACACTC	7218-7242	3D	-	In this study

[1] Corresponding to the position of QT2013 genome (Genbank accession no. KJ463384). Small letters indicated the additional universal sequence for RACE PCR. Abbreviations: base pair, bp.

Table S2 Reference sequence used in this study

Strain	Year	Country	Accession No.
V13	1958[1]	UK	AF406813
C-6	2015	India	KY053835
csh	2009	China	HQ875059
YC2011	2011	China	JX286666
JD2011	2011	China	KF539414
QT2013	2013	China	KJ463384
HuN1	2015	China	KX354740
HuN2	2015	China	KX354741
HuN3	2015	China	KX354742
HuN4	2015	China	KX354743
HuN5	2016	China	MF440633
HuN6	2016	China	MF440634
HuN7	2016	China	MF440635
HuN8	2016	China	MF440636
HuN9	2016	China	MF440637
HuN10	2016	China	MF440638
HuN11	2016	China	MF440639
HuN12	2016	China	MF440640
HuN13	2016	China	MF440641
HuN14	2016	China	MF440642
HuN15	2016	China	MF440643
HuN16	2016	China	MF440644
HuN17	2016	China	MF440645
HuN18	2017	China	MF440646
HuN19	2017	China	MF440647
HuN20	2017	China	MF440648
HuN21	2017	China	MF440649
HuN22	2017	China	MF440650
HuN23	2017	China	MF440651
HuN24	2017	China	MF440652
HuN25	2014	China	MF440653
HuN26	2017	China	MF440654
HuN27	2017	China	MF440655

HuN28	2017	China	MF440656
HuN29	2017	China	MF440657
HuN30	2017	China	MF440658
HuN31	2017	China	MF440659
HuN32	2017	China	MF440660
HuN33	2014	China	MF440661
PSV-A2	2016	China	MG732813
PSV-C	2016	China	MG732814
JXXY-a2	2017	China	MH626634
JXXY-C	2017	China	MH626635
KS04105	2004	South Korea	KJ821019
KS05151	2005	South Korea	KJ821020
KS055217	2005	South Korea	KJ821021
IA33375	2015	USA	KX574284
ISU-SHIC	2016	USA	KX810827
Jpsv447	2009	Japan	LC326556
Jpsv1315	2009	Japan	LC326555
L00798-K11 14-02	2014	Germany	LT900497
OPY-1	2017	France	MH513612

[1] Reported in 1958

Table S3 Summary of the history and prevalence of PSV infection in pigs in Zambia

				No. of positive/No. of tested sample (%)			
			Number	Suckling stage (0-3 weeks)		Fattening stage (4-12 weeks)	
Farm	District	Sampling date	of sample	No diarrhea	Diarrhea	No diarrhea	Diarrhea
A	Lusaka	7/January/2018	24	2/5 (40)	5/12 (42)	-	7/7 (100)
		14/June/2018	15	-	4/4 (100)	2/2 (100)	9/9 (100)
B	Chilanga	25/January/2018	4	-	-	-	4/4 (100)
		10/July/2018	15	-	-	9/9 (100)	6/6 (100)
		4/December/2018	11	0/1 (0)	1/2 (50)	1/1 (100)	6/7 (86)
C	Kafue	13/February/2018	15	-	0/5 (0)	-	8/10 (80)
		8/June/2018	16	-	2/7 (29)	7/8 (88)	1/1 (100)
		16/August/2018	27	-	2/10 (20)	12/13 (92)	3/4 (75)
D	Chibombo	2/March/2018	4	-	-	-	4/4 (100)
E	Lusaka	17/July/2018	10	-	-	4/4 (100)	6/6 (100)
		20/December/2018	6	1/1 (100)	-	4/4 (100)	1/1 (100)
Total			147	3/7 (42.9)	14/40 (35.0)	39/41 (95.1)	55/59 (93.2)

Table S4 Deduced amino acid sequence lengths of encoded proteins in Zambian PSVs

Isolate	Lengths of encoded proteins (aa)											
	L	VP4	VP2	VP3	VP1	2A	2B	2C	3A	3B	3C	3D	Polyprotein
PSV-20	84	53	238	234	294	226	105	332	100	22	182	462	2332
PSV-21	84	53	238	234	285	226	105	332	100	22	182	462	2323
PSV-22	84	53	238	234	285	226	105	332	100	22	182	462	2323
PSV-23	84	53	238	234	285	226	105	332	100	22	182	462	2323
PSV-26	84	53	238	234	293	226	105	332	100	22	182	462	2331
PSV-46	84	53	238	234	285	226	105	332	100	22	182	462	2323

Abbreviation: amino acid, aa.

Table S5 Identity comparison of complete ORFs between PSVs

Strain	Country	Nucleotide and deduced amino acid identities (%)1:											
		PSV-20		PSV-21		PSV-22		PSV-23		PSV-26		PSV-46	
		nt	aa	nt	aa	nt	aa	nt	aa	nt	aa	nt	aa
PSV-20	Zambia												
PSV-21	Zambia	89.5	95.3										
PSV-22	Zambia	89.5	95.3	99.5	99.7								
PSV-23	Zambia	89.6	95.1	99.1	99.4	99.0	99.4						
PSV-26	Zambia	92.5	98.2	86.7	94.5	86.7	94.5	86.7	94.3				
PSV-46	Zambia	88.5	96.6	85.6	93.8	85.6	93.8	85.7	93.7	88.2	96.7		
HuN1	China	88.1	95.9	87.3	95.8	87.3	95.8	87.3	95.6	86.9	95.7	86.6	94.9
HuN2	China	89.2	97.3	86.9	94.0	86.9	94.0	87.0	93.8	88.5	97.5	87.8	95.8
HuN3	China	89.4	97.9	86.2	94.2	86.2	94.2	86.3	94.1	89.1	98.1	88.2	**96.5**
HuN4	China	**90.6**	**98.3**	87.1	94.4	87.1	94.4	87.2	94.4	89.3	98.1	**88.3**	96.5
HuN9	China	87.4	95.7	87.4	95.6	87.4	95.6	87.5	95.4	87.1	95.4	86.8	95.0
HuN12	China	86.3	94.6	**88.7**	**97.7**	**88.7**	**97.7**	**88.8**	**97.6**	86.0	94.2	85.7	94.2
HuN17	China	87.3	95.4	86.8	95.5	86.8	95.4	86.8	95.2	86.7	95.2	86.5	94.4
HuN23	China	89.7	98.1	86.2	94.4	86.2	94.3	86.3	94.1	89.2	97.9	88.1	96.2
HuN27	China	87.8	95.4	87.0	95.6	87.1	95.5	87.1	95.4	87.2	95.1	86.7	94.5
QT2013	China	87.9	95.6	87.4	96.0	87.4	95.9	87.5	95.7	87.0	94.9	86.8	94.5
PSV-A2	China	89.9	98.1	86.5	94.3	86.4	94.3	86.7	94.2	**89.6**	**98.5**	87.8	96.4
Jpsv447	Japan	87.5	97.1	84.4	93.3	84.4	93.2	84.4	93.1	87.5	97.3	86.6	95.6
ISU-SHIC	USA	84.5	93.1	86.3	96.1	86.3	96.1	86.3	95.9	84.0	93.2	83.3	92.6
C-6	India	84.3	93.6	83.9	93.5	83.9	93.4	83.8	93.3	84.4	93.9	84.4	93.9
V13	UK	83.9	93.2	84.1	94.0	84.1	94.0	84.1	93.9	83.9	93.6	84.1	93.4

[1]The highest identities between the Zambian PSVs and previously reported PSVs are shown in bold face.

Abbreviation: nucleotide sequence identity, nt; deduced amino acid identity, aa.

Table S6 Identity comparison of encoded proteins among Zambian PSVs

Protein	Range of deduced amino acid identities (%):
L	94.0-100
VP4	92.4-100
VP2	94.5-100
VP3	88.0-100
VP1	78.9-99.6
2A	88.9-99.1
2B	98.0-100
2C	97.8-100
3A	97.0-100
3B	90.9-100
3C	97.8-100
3D	98.9-100

Table S7 Predicted recombination in Zambian PSVs, PSV-20, PSV-26, and PSV-46

Recombinant	Predicted potential parents		Break point		P value of recombination analyses using each bioinformatics method						
	Major	Minor	Beginning	Ending	RDP	GeneConv	BootScan	MaxChi	Chimera	SiScan	3Seq
PSV-20	JPSV447	PSV-21	2790	270	1.458×10^{-36}	7.602×10^{-25}	1.672×10^{-39}	8.804×10^{-33}	1.182×10^{-06}	3.516×10^{-42}	2.753×10^{-12}
	HuN17	Unknown	2165	2532	1.319×10^{-3}	-	5.951×10^{-1}	1.042×10^{-2}	1.263×10^{-2}	7.607×10^{-6}	-
PSV-26	PSV-22	Jpsv447	310	2749	4.292×10^{-22}	2.412×10^{-11}	1.549×10^{-18}	1.511×10^{-18}	3.481×10^{-22}	2.532×10^{-23}	2.753×10^{-12}
PSV-46	JD2011	Unknown	1974	Undetermined	2.361×10^{-3}	1.224×10^{-2}	7.745×10^{-4}	1.294×10^{-3}	5.976×10^{-4}	2.778×10^{-7}	8.158×10^{-6}
	JXXY-C	Unknown	2708	4852	7.020×10^{-11}	1.087×10^{-9}	7.281×10^{-14}	5.082×10^{-8}	1.279×10^{-10}	6.055×10^{-15}	8.261×10^{-12}
	Unknown	HuN26	Undetermined	6101	3.892×10^{-5}	-	4.452×10^{-5}	1.977×10^{-7}	6.676×10^{-7}	2.328×10^{-7}	6.383×10^{-8}

- Not predicted

Table S8 Amino acid residue unique to the Lineage 1

Protein	Amino acid position	Amino acid	
		Leneage 1	Leneage 2 and 3
VP4	45	G	N
VP4	46	M	A
VP4	47	V	M
VP4	51	T	S
VP2	18	V	A
VP2	45	A	G
VP2	58	S	A
VP2	69	R	Q
VP2	72	D	N
VP2	124	I	V
VP2	141	Q	E
VP2	159	P	T
VP2	209	A	D
VP2	227	Q	M
VP3	7	I	V
VP3	29	Q	E
VP3	33	N	G
VP3	56	S	R
VP3	58	S	N
VP3	59	T	V
VP3	62	T	A
VP3	63	N	S
VP3	64	R	G
VP3	65	I	V
VP3	71	I	V
VP3	75	D	N
VP3	77	S	T
VP3	78	S	A
VP3	80	M	I
VP3	81	V	A
VP3	90	S	E or G
VP3	93	S	V or A
VP3	178	L	V
VP3	179	T	I or N
VP3	211	A	V
VP3	216	S	A
VP1	5	E	D
VP1	13	S	K
VP1	14	V	T
VP1	22	R	K or M
VP1	25	Q	K or R
VP1	88	G	D
VP1	89	T or I	K
VP1	96	T	Q
VP1	104	Q	N
VP1	114	G	S
VP1	136	M	L or V
VP1	141	A	P
VP1	142	S	A
VP1	143	K	A

VP1	145	S	K
VP1	147	V	L
VP1	153	F	Y
VP1	156	N	H
VP1	159	A	V
VP1	162	T	D or N
VP1	163	T	R
VP1	165	R	D or N
VP1	166	T	S
VP1	167	Q	R
VP1	168	I	V
VP1	183	N	D
VP1	184	A	C
VP1	208	Y	N or T
VP1	211	N	Q or R
VP1	212	S	N
VP1	213	A	G
VP1	228	S	A
VP1	231	M	L
VP1	236	D	V or I
VP1	238	D	A or N
VP1	239	A	E
VP1	240	T	V
VP1	241	T	S
VP1	243	A	S
VP1	246	A	V
VP1	265	S	A
VP1	273	R	V
VP1	274	L	Q
VP1	278	Q	T
VP1	280	F	Y
2A	13	D	E
2C	11	V	T or I or M
3B	8	R	K
